# Induction of Nitric Oxide and Its Role in Otitis Media

**DOI:** 10.3390/antiox14030327

**Published:** 2025-03-10

**Authors:** Yeon Ju Oh, Jae Min Lee, Joon Hyung Yeo, Sung Soo Kim, Seung Geun Yeo

**Affiliations:** 1 Department of Medicine, College of Medicine, Kyung Hee University Medical Center, Seoul 02447, Republic of Korea; 5duswn1203@khu.ac.kr; 2 Department of Otorhinolaryngology Head and Neck Surgery, Kyung Hee University School of Medicine, Kyung Hee University Medical Center, Seoul 02447, Republic of Korea; sujaesa@hanmail.net; 3 Public Health Center, Danyang-gun 27010, Chungcheongbuk-do, Republic of Korea; joonhyungyeo@gmail.com; 4 Department of Biochemistry and Molecular Biology, College of Medicine, Kyung Hee University, Seoul 02447, Republic of Korea; sgskim@khu.ac.kr; 5 Department of Precision Medicine, Graduate School, Kyung Hee University, Seoul 02447, Republic of Korea; 6 Department of Convergence Medicine, College of Medicine, Kyung Hee University, Seoul 02447, Republic of Korea

**Keywords:** otitis media, cholesteatoma, nitric oxide

## Abstract

Although many studies have investigated the expression and role of nitric oxide (NO) in various diseases, it remains unclear whether NO has a beneficial or detrimental impact on otitis media. This review examines the literature on the expression and role of NO in different forms of otitis media, including acute otitis media, otitis media with effusion, chronic otitis media, and cholesteatomatous otitis media. Of the 22 studies reviewed, 18 reported that NO induces or exacerbates otitis media, whereas two studies suggested that NO may aid in its treatment. Factors contributing to these conflicting results include the type of otitis media studied, the duration of the condition, the types of samples collected, and the specific type of NO synthase targeted. Comprehensive analysis indicates that NO expression may be higher in chronic otitis media than in acute forms and is more pronounced in patients with cholesteatoma than in those without it. Although these findings suggest that NO inhibitors could potentially aid in the treatment of otitis media, NO could also aid in its treatment by inhibiting bacterial infections. Despite the dual potential of NO, current evidence suggests a strong association between NO and the pathophysiology of otitis media.

## 1. Introduction

### 1.1. Otitis Media

Otitis media refers to an inflammatory disease occurring in the middle ear. It is classified based on the duration of the condition: acute otitis media if it lasts less than 3 weeks, subacute otitis media if it lasts between 3 weeks and 3 months, and chronic otitis media if it persists for more than 3 months. Acute otitis media encompasses all acute inflammatory phenomena occurring in the middle ear cavity. While most cases resolve without sequelae, some cases may recur or persist as recurrent otitis media or otitis media with effusion, and eventually manifest as chronic otitis media if inflammation in the middle ear cavity is not fully resolved. The precise factors leading to the transition from acute infection in the middle ear and the mastoid to chronic inflammation have not been fully elucidated [1,2,3].

Otitis media with effusion is defined as the presence of effusion in the middle ear cavity without symptoms or signs of acute inflammation and is a common cause of hearing loss in children. It is primarily attributed to adenoid hypertrophy, allergies, sinusitis, and upper respiratory infections. Without proper diagnosis and treatment, otitis media with effusion in young children can lead to hearing loss, affecting language acquisition, communication skills, behavior, and learning. Most cases improve spontaneously throughout observation and follow-up; however, surgical intervention, such as tympanostomy tube insertion, may be necessary for patients without improvement. In young children, the Eustachian tube is underdeveloped, shorter, and more horizontally positioned, making it less effective at protecting the middle ear from nasopharyngeal bacterial contamination and pressure changes [4,5,6].

Chronic otitis media is a persistent inflammatory disease of the middle ear or mastoid and is typically considered a complication of frequent acute otitis media episodes in childhood. It can also result from various traumas or chronic perforations of the tympanic membrane following tympanostomy tube placement. The tympanic membrane may also exhibit thickening, atrophy, or myringosclerosis with tympanosclerosis around the ossicles. Chronic otitis media leads to tympanic membrane perforation, irreversible changes, and alterations in the mucosa or bony structures of the middle ear cavity and the mastoid due to recurrent inflammation. The degree of surrounding tissue changes and hearing loss varies with the severity and duration of inflammation, and untreated cases can lead to various intracranial and extracranial complications [7,8,9].

Cholesteatomatous otitis media presents as an epidermal cyst or keratinized epithelial form in the middle ear and the mastoid. It consists of a matrix of squamous epithelium surrounding the accumulated keratin and a perimatrix of inflammatory granulation tissue, characterized by progressive enlargement and destruction of the surrounding bony structures in the temporal bone. Although it primarily occurs in the middle ear and the mastoid, in rare cases, cholesteatoma can extend to the petrous bone or the external auditory canal, either primarily or secondarily. When keratin accumulates within the cyst or the sac, the cholesteatoma matrix grows towards the basement membrane, and if infection accompanies it, enzymes such as collagenase are secreted from the granulation tissue, eroding the adjacent anatomical structures and bone. Cholesteatomatous otitis media is not amenable to medical treatment and requires surgical removal, but it is relatively prone to recurrence [10,11,12].

### 1.2. Nitric Oxide

Nitric oxide (NO) is a free radical composed of one nitrogen atom and one oxygen atom with an unpaired electron, possessing oxidative properties. Being uncharged, it can freely diffuse across cells and cell membranes. These properties enable NO to function as an important signaling molecule in biological systems. Additionally, due to its strong toxicity, NO acts as a mediator of cytotoxicity in host defense mechanisms [13]. NO is synthesized from L-arginine (an amino acid) by nitric oxide synthase (NOS), requiring NADPH and O_2_ and generating citrulline alongside NO. There are three known types of NOS: type I (neuronal NOS, nNOS), type II (inducible NOS, iNOS), and type III (endothelial NOS, eNOS). Type I NOS (nNOS) has a molecular weight of 160 kDa; it is initially identified in neurons, and also exists in a cytosolic (free, soluble) form. Type II NOS (iNOS) has a molecular weight of 130 kDa; it is first identified in macrophages, and also exists cytosolically. Type III NOS (eNOS) has a molecular weight of 133 kDa; it is first identified in endothelial cells, and is membrane-bound. Type I and III NOS, nNOS and eNOS, are generally constitutively expressed and are referred to as constitutive NOS (cNOS), whereas iNOS is inducible, being transcribed and expressed only upon specific stimuli. Under normal conditions, NO is synthesized by type I and III NOS, with synthesis regulated post-translationally by Ca²⁺/calmodulin. Under abnormal conditions, NO is synthesized by type II NOS, with its induction and production activated by cytokines such as endotoxin, IL-1, TNF-α (tumor necrosis factor-α), TNF-β (tumor necrosis factor-β), and lymphotoxin, rather than by Ca²⁺/calmodulin [14]. 

NO operates via two mechanisms. The first is through a cyclic GMP (cGMP)-dependent pathway, where NO binds to the heme of soluble guanylate cyclase (sGC), converting GTP to cGMP. Then, cGMP activates cGMP-dependent protein kinase, cGMP-regulated phosphodiesterase, and cyclic nucleotide-gated ion channels, resulting in physiological functions such as vasodilation, neurotransmission, inhibition of platelet aggregation, and smooth muscle relaxation. The second mechanism is a cGMP-independent pathway, where NO reacts with O_2_, superoxide (O_2_^−^) thiols, or transition metals (e.g., zinc) to exert its effects. Constitutive NOS (cNOS: nNOS, eNOS) generates small amounts of NO over short periods, primarily involved in homeostatic regulation such as vasoconstriction and neurotransmission. In contrast, iNOS releases large quantities of NO over extended periods, exerting cytostatic or cytotoxic effects via both cGMP-dependent and cGMP-independent pathways Table 1.

Under normal conditions, small amounts of NO (10–12 moles, picomole) are released for short durations, activating sGC to enhance cGMP production from GTP, facilitating processes such as neurotransmission, vascular smooth muscle relaxation, and inhibition of platelet aggregation. NO is inactivated by forming nitrite (NO₂⁻) through a reaction with O₂ or by interacting with iron in proteins to regulate iron homeostasis. Alternatively, NO reacts with thiols (R–SH) to form stable nitrosothiol (R–S–NO) for transport or nitrosylates thiol proteins, influencing axon terminal remodeling. Under abnormal conditions, large quantities of NO (10–9 moles, nanomole) are continuously released with elevated intracellular O₂⁻ levels and depleted thiol pools. NO reacts with O₂⁻ to form peroxynitrite, subsequently generating highly toxic hydroxyl radicals. NO can inhibit enzymatic activity by reacting with Fe–S or R–SH groups and cause irreversible ADP-ribosylation by nitrosylating GADPH (glyceraldehyde-3-phosphate dehydrogenase). NO also induces DNA deamination, activating PARS (polyADP-ribose synthetase), leading to cellular embolism, energy depletion, mutagenesis, and ultimately cell death [15,16].

**Figure 1:**
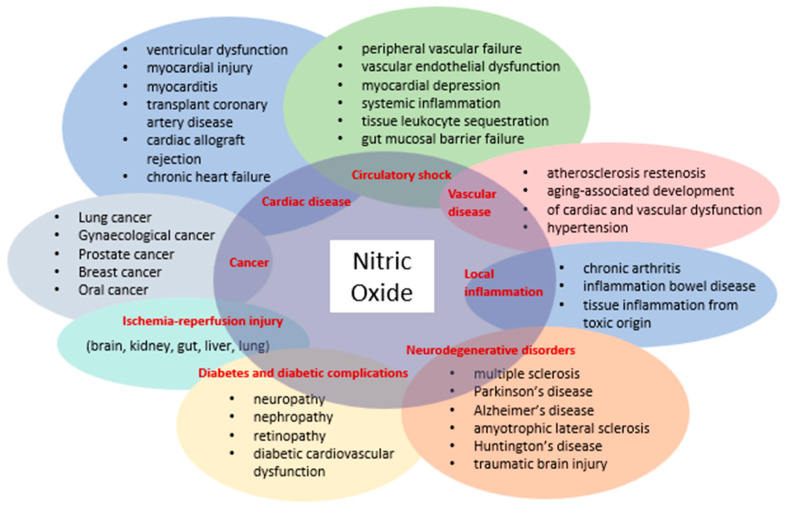
Diseases involving NO and peroxynitrite.

The discovery that mammalian cells can synthesize the free radical NO has spurred extensive research across all fields of biology and medicine. Initially introduced as an endothelium-derived relaxing factor, NO is now recognized as a fundamental signaling molecule regulating cellular function and as a potent mediator of cell damage under various conditions. Most NO-induced cytotoxicity arises from peroxynitrite, formed by the reaction of NO with a superoxide anion, which interacts with lipids, DNA, and proteins through direct oxidative reactions or indirect radical-mediated mechanisms. Peroxynitrite production in vivo is significantly associated with conditions such as stroke, myocardial infarction, chronic heart failure, diabetes, circulatory shock, chronic inflammatory diseases, cancer, and neurodegenerative disorders (Figure 1) [17].

## 2. Research Methods

**Figure 2:**
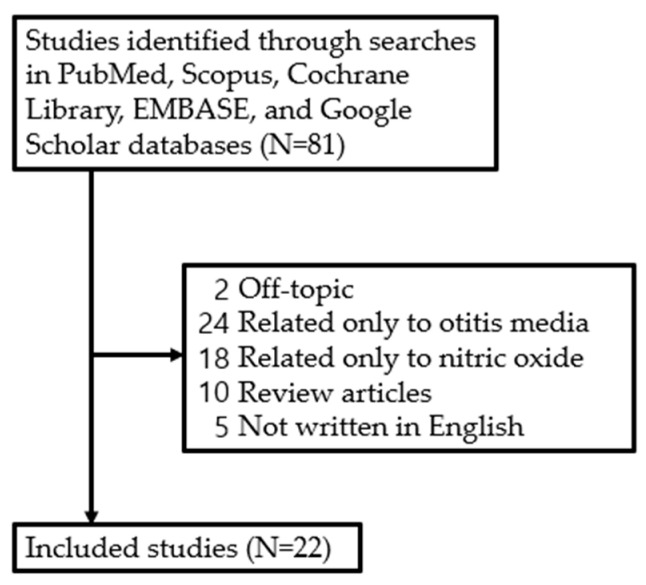
Review flow diagram.

Although the role of NO in various diseases has been studied, there has not been a systematic study on the expression and function of NO in the development of otitis media. Therefore, studies examining recovery from facial nerve injury and the effects of NO published between January 1994 and March 2024 were retrieved from five electronic databases—PubMed, Scopus, Cochrane Libraries, Embase, and Google Scholar—by one of the authors (J.H.Y) based on the search terms ‘otitis media’ and ‘nitric oxide’. The literature search focused on studies published in English, including (1) prospective or retrospective studies on NO in otitis media and (2) studies involving humans and animals. However, studies were excluded if they were (1) unpublished data, (2) review articles, (3) grey literature, (4) case reports, or (5) duplicates. As a result, a review of the literature was conducted on 22 studies, excluding 59 out of a total of 81 studies retrieved (Figure 2).

## 3. Studies on the Role of NO in Otitis Media

Research on the expression and role of NO in AOM, OME, COM with or without cholesteatoma, and COM with or without tympanosclerosis has utilized direct measurement methods of NO as well as indirect methods using NO-related substances or NO stimulants or inhibitors. The indirectly utilized substances have included NOS (iNOS, eNOS, nNOS), peroxynitrite, 3-nitrotyrosine, S-nitroso-N-acetylpenicillamine (SNAP), malondialdehyde (MDA), 4-hydroxynonenal, myeloperoxidase, N(G)-nitro-L-arginine methyl ester (L-NAME), glutathione peroxidase (GPx), catalase (CAT), superoxide dismutase (SOD), steroids (dexamethasone, glucocorticoids), and tacrolimus [18].

### 3.1. Nitric Oxide (NO)

The expression and increase in NO in otitis media have been reported to be related to the development and pathogenesis of otitis media. In a study directly measuring NO in otitis media with effusion, 50 patients with OME were compared in terms of NO, as well as the percentages of CD4+ and CD8+ T lymphocytes, CD4+/CD8+ ratio, IL-2, IL-4, IL-6, and immunoglobulin E (IgE) in peripheral blood and middle ear effusion (MEE) with 50 healthy children as the control group. The levels of CD4+ and CD8+ in peripheral blood, CD4+/CD8+ ratio, IgE, and NO were significantly higher in the experimental group than in the control group (*p* < 0.01). The levels of IL-2, IL-6, IgE, and NO in MEE were significantly higher than those in peripheral blood in the experimental group (*p* < 0.01), suggesting that the increase in NO levels is involved in the pathogenesis of otitis media with effusion [19] (Table 2). 

Conversely, there are reports that NO expression upon antibiotic use enhances the bactericidal effect against *Streptococcus pneumoniae* (*S. pneumoniae*), aiding in otitis media treatment. *S. pneumoniae* is one of the main pathogens of otitis media, a common infection in children and a major cause of antibiotic prescriptions. Biofilm formation is a significant phenotype contributing to antibiotic resistance and persistence of *S. pneumoniae* in chronic or recurrent OM. It was hypothesized that adding low concentrations of NO to the *S. pneumoniae* biofilm would enhance antibiotic efficacy and exert direct antimicrobial effects at high concentrations. However, the study found that, unlike many other bacterial species, low concentrations of NO did not induce *S. pneumoniae* biofilm dispersion. Instead, pneumococcal killing was enhanced when in vitro biofilms and ex vivo adenoid tissue samples (reservoirs of *S. pneumoniae* biofilm) treated with low concentrations of NO were combined with amoxicillin–clavulanic acid, an antibiotic commonly used for chronic OM treatment. Thus, low concentrations of NO appear to regulate pneumococcal metabolism, suggesting a novel therapeutic approach to reduce antibiotic resistance in pneumococcal biofilms [37] (Table 3). 

The finding that NO exhibits antibacterial effects by inhibiting biofilm formation suggests that NO-based therapy, consisting of combinations of antibiotics and NO donors, may have potential in the treatment of otitis media. Exogenous NO at picomolar to nanomolar concentrations can trigger biofilm dispersion. Moreover, endogenous NO could promote the conversion from sessile to planktonic mode. The effects of NO are exerted through several antimicrobial mechanisms, such as biofilm dispersion, which hinders the development of bacterial resistance to NO. Additionally, NO may limit the recycling of Fe^2+^, which would otherwise lead to macromolecular damage from hydroxyl radicals generated by the Fenton reaction [38], a process that involves the generation of hydroxyl radicals from ferrous iron and hydrogen peroxide [39].

**Table 3:** Studies claiming that NO aids in the treatment of otitis media.

Author [Reference]	Study Design	Species and/or Sample	Types of Otitis Media	Detection Method	Target Gene(s) or Pathway(s) Associated with NOS	Results/Conclusions
Allan et al. (2016) [37]	Biofilm experiment, human study	11 pediatric patients	Otitis media caused by *S. pneumoniae*	CLSM, SEM, iTRAQ labeling, mass spectrometry, peak list generation, database searching	NO	Treatment with the NO donor SNP reduced the viability of planktonic cells and decreased the cell population within in vitro biofilms. Adjunctive NO treatment of in vitro pneumococcal biofilms increased the effectiveness of antibiotics. The combination of antibiotics and NO treatment improved the ex vivo eradication of *S. pneumoniae* on adenoid tissue. These findings imply that at lower concentrations, NO disrupts the metabolism of pneumococcal biofilms, while at higher concentrations, NO becomes toxic to *S. pneumoniae*. Targeted adjunctive NO treatment could be a promising novel therapy for reducing biofilm tolerance by pneumococci.
Granath et al. (2010) [40]	Human study	12 children with OME and 14 children with adenoid hypertrophy (control)	Otitis media with effusion	Real time PCR, immunohistochemical staining of adenoid tissue	iNOS, eNOS	The children with OME showed lower levels of iNOS compared to the controls without middle ear disease, while no difference was observed for eNOS. These proteins were primarily located in association with the surface epithelium. The local induction of iNOS in adenoids may play a crucial role in preventing the development of OME.

Abbreviation: OME, otitis media with effusion; PCR, polymerase chain reaction; SNP, sodium nitroprusside dehydrate; CLSM, confocal laser scanning microscopy; SEM, scanning electron microscopy; iTRAQ, isobaric tags for relative and absolute quantitation.

The Fenton reaction, however, can also contribute to the formation of free radicals in the body, leading to pathological outcomes. Iron and hydrogen peroxide can oxidize a wide range of substrates and cause biological damage. The Fenton reaction is complex and capable of generating both hydroxyl radicals and higher oxidation states of iron [41]. The ability of NO to exhibit antibacterial effects and suppress infection when used with antibiotics does not exclude the possibility that NO has pathological effects in the body. Additional experimental results supporting the latter have been observed in patients with otitis media.

### 3.2. NO Metabolites and NO Donors

In a study involving 55 patients with otitis media with effusion (OME), effusion was classified as mucoid otitis media (MOM), serous otitis media (SOM), and purulent otitis media (POM) based on effusion characteristics, and levels of NO metabolites were compared. Concentrations of NO metabolites were highest in MOM, followed by SOM and POM, suggesting that NO may mediate mucin secretion and play a crucial role in the pathogenesis of OME [20,38,39,41].

Research using NO donors included both in vitro and in vivo studies. In an in vitro study using HT-29MTX goblet cells, the NO donor isosorbide dinitrate (ISDN) was cultured at concentrations of 0.01, 0.1, 0.5, 1, and 2 mM, consistently increasing mucus production compared to the control group. The maximum mucus production increased by 35% in the group cultured with 1 mM ISDN for 1 h compared to the control group. Mucus production increased by 12% in the 0.1 mM ISDN group and by 45% over baseline in the 2 mM ISDN group. NO donation by ISDN increased mucus production, which was related to concentration and time, helping to explain mucus secretion mechanisms in OME [21].

In an in vivo study using chinchillas, S-nitroso-N-acetylpenicillamine (SNAP), LPS (lipopolysaccharide), and LPS + SNAP were injected into the superior bullae to induce otitis media, and mucus concentration and histological changes were compared. Mucus concentration was highest in the LPS + SNAP group and lowest in the SNAP-only group. The greatest mucosal thickening and inflammation were observed in the LPS + SNAP group in histopathological studies, indicating that NO contributes to the pathogenesis of mucoid otitis media [22].

### 3.3. Nitric Oxide Synthase (NOS)

**Figure 3:**
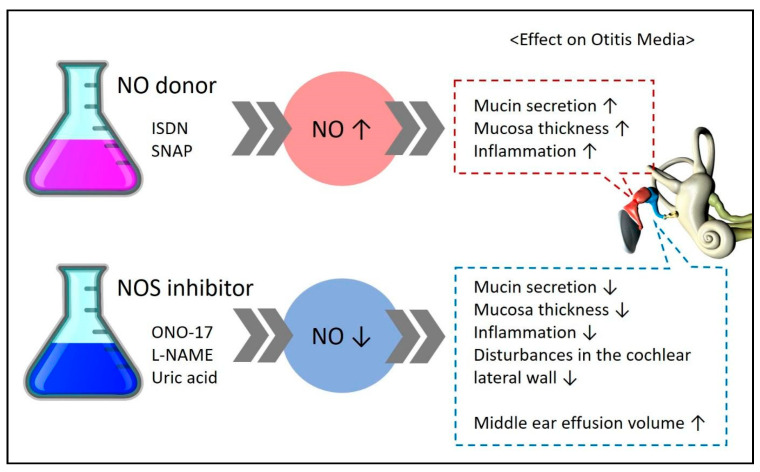
Experimental results demonstrating the effects on disease upon administration of an NO donor/NOS inhibitor in experimental groups. ↑ means increase, ↓ means decrease.

Some studies have measured NOS (iNOS, eNOS, nNOS), an enzyme essential for NO formation, instead of directly measuring NO to understand its expression and role (Figure 3).

#### 3.3.1. NOS

Assuming that microbial infection in the middle ear cavity of OME patients induces the expression of toll-like receptors (TLRs), cytokines, and NO, a study without a normal control group divided participants into otitis-prone and non-otitis-prone groups to compare TLR, cytokine, and NOS mRNA levels in middle ear effusion using real-time polymerase chain reaction. TLR-1, -2, -4, -5, -6, -9; IL-6, -8, -10, -12; IFN-γ; TNF-α; and NOS mRNA expression was measured in effusions from both otitis-prone and non-otitis-prone groups. TLR-2, -4, -6, and -9 mRNA expression was significantly lower in the otitis-prone group (*p* < 0.05), and TLR, cytokine, and NOS mRNA expression levels were higher in the culture-positive patients. Thus, NOS was closely related to OME and involved in its pathogenesis [23].

#### 3.3.2. iNOS

Among the studies focusing on different types of NOS, researchers have primarily used iNOS. In four studies—two involving animals and two involving humans—iNOS expression was related to otitis media pathogenesis. However, one human study yielded opposing results, suggesting that iNOS expression in adenoid samples, rather than in middle ear effusion or inflammatory tissue, may prevent OME development.

In an experiment with 16 healthy rabbits, gastric contents were injected into the middle ear for 20 days in the experimental group, while saline was injected in the control group, with observations on the 27^th^ day. The experimental group showed thickened lamina propria and epithelium, increased active fibroblasts, collagen fibers, inflammatory cells, and vascular dilation. VEGF, iNOS, IL-1β, and IL-17 expression were significantly increased in the experimental group compared to the control group (*p* = 0.018, *p* = 0.010, *p* = 0.002, *p* = 0.002, respectively), showing that middle ear inflammation induced by gastroesophageal reflux was related to increased expression of VEGF, IL-1β, IL-17, and iNOS [24].

A similar study using *S. pneumoniae*, the most common strain of acute otitis media, instead of gastroesophageal reflux, showed comparable results. Comparing the *S. pneumoniae*-injected experimental group to the saline-injected control group in rats, significant upregulation of IL-1α, IL-1β, IL-6, IL-10, tumor necrosis factor alpha, and iNOS gene expression was observed in the experimental group (*p* < 0.05). These results indicated that iNOS increase was related to the pathogenesis of acute otitis media in rats [25]. Human studies have shown similar results to animal studies.

A comparative histological study of 9 children with otitis media with effusion and 11 patients with chronic otitis media and tympanosclerosis showed a higher expression of macrophages, B cells, and IL-6 in the otitis media with effusion group. Expression of iNOS was higher in the patients with chronic otitis media and tympanosclerosis than in the otitis media with effusion group. IL-6 and iNOS were mainly stained in surface cells, while macrophages and B cells stained deeper in the tissue, connective tissue, or around sclerotic lesions. These results indicated that iNOS was more frequently observed in patients with established tympanosclerosis, i.e., at later stages of the disease [26].

Research on the antibiotics used to treat otitis media has also focused on the NOS expression and role. An experimental guinea pig otitis media model was used to evaluate the effects of macrolide antibiotics (erythromycin, azithromycin, roxithromycin, and clarithromycin), showing that they increased NOS activity and decreased XO activity and MDA levels, key indicators of oxidative stress [42].

Contrary to the existing studies, one study reported decreased iNOS. Adenoid samples from the children with OME showed a lower iNOS expression compared to samples from the children without middle ear disease. It was suggested that local induction of iNOS in adenoids might be important in preventing OME development [40]. However, considering that the study involved adenoids rather than inflammatory cells or effusion from the middle ear, the direct target organ of otitis media, there are issues in comparing these results with other studies.

#### 3.3.3. Polymorphisms

One study investigated polymorphisms in eNOS. Genetic analysis using PCR (polymerase chain reaction) was conducted on blood samples from 89 patients diagnosed with otitis media with effusion and 85 age- and gender-matched healthy controls. The genetic analysis revealed no significant differences in eNOS Glu298Asp polymorphism (G/G, G/T, T/T) between the patients and the controls. However, a significant relationship was found when comparing allele distributions between the groups (*p* = 0.037), suggesting that the G allele might predispose individuals to OME development [43] (Table 4).

#### 3.3.4. NO and NOS

A study measuring both NO and NOS in the guinea pigs with otitis media with effusion (*n* = 6) and the controls (*n* = 6) found that NO synthase activity and NO levels in the otitis media with effusion group were significantly higher than in the control group. A significant positive correlation was observed between NO synthase activity and NO levels in the otitis media with effusion group. Thus, increased NO levels might play a significant role in cell and tissue damage caused by experimental otitis media with effusion [27].

### 3.4. NO Stimulants or Inhibitors, NO Metabolites, NO Biomarkers

#### 3.4.1. Steroids

Various arginine analogues used as NO inhibitors include L-NMMA (Nω-monomethyl-L-arginine), L-ADMA (NωNω-dimethyl-L-arginine), L-NAME (Nω-nitro-L-arginine methyl ester), L-NNA (Nω-nitro-L-norarginine), L-NA (NG-nitro-L-arginine), Nω-amino-L-arginine, NOLA, LNIO (Nω-imminoethyl-L-ornithine), L-canavanine, D-arginine, and L-glutamate. However, the steroids widely used clinically for various diseases have also been studied as NO inhibitors. In a study with Sprague–Dawley rats, LPS was inoculated into the right middle ear cavity, and 30 min later, dexamethasone, a NOS inhibitor, or PBS was administered intratympanically. Additionally, prostaglandin E (1) was applied locally to the round window membrane of the right ear, and changes in the cochlear blood flow were measured. The increase in the cochlear blood flow following prostaglandin application was higher in the group that received the NOS inhibitor. Furthermore, vascular tissue changes were less severe in the rats treated with dexamethasone or the NOS inhibitor. This study demonstrated that intratympanic administration of dexamethasone or a NOS inhibitor is effective in treating cochlear lateral wall damage due to acute otitis media [28].

Another study aimed to compare the effects of different steroid formulations, using dexamethasone, fluticasone propionate, and rimexolone to determine if they could reduce the NO concentration in middle ear effusion (MEE). In 53 chinchillas, LPS was repeatedly administered to the middle ear, and MEE was collected after 96 h. All three glucocorticoids at 0.1% decreased the NO concentration in the middle ear, but only fluticasone propionate showed a significant reduction. At a 1.0% concentration, all three steroids significantly reduced NO levels. This study suggests that glucocorticoid treatment reduces the NO concentration in MEE, potentially protecting the ear from NO-induced sensorineural hearing loss (SNHL) [29].

#### 3.4.2. Steroids and Tacrolimus

Tacrolimus, a secondary macrolide product with immunosuppressive and antifungal activity, is used not only to suppress immune rejection in organ transplant patients, but also to treat autoimmune diseases such as atopy. Tacrolimus is a potent and promising immunosuppressant, showing 10 to 100 times the immunosuppressive effect of cyclosporin, the most widely used immunosuppressant, with fewer side effects such as hirsutism, gingival hyperplasia, or hypertension. In a study on rats with acute otitis media induced by *S. pneumoniae*, the subjects were randomly assigned to control or treatment with saline, aminoguanidine, anisomycin, dexamethasone, ketorolac, L-N (G)-nitroarginine methyl ester, methylprednisolone, mycophenolic acid, pentoxiphylline, tacrolimus, or WEB2086, totaling 12 groups. After 48 h, IL-6, iNOS, and MCP-1 were significantly increased in the middle ear mucosa due to infection. MCP-1 was reduced in most treatment groups, with tacrolimus and dexamethasone particularly reducing IL-6, iNOS, and MCP-1 [30].

#### 3.4.3. L-LAA

In one study, guinea pigs were systemically immunized, with one ear challenged with an antigen only and the opposite ear challenged with both an antigen and the potent NOS inhibitor N(G)-amino-L-arginine (L-NAA). At 24, 48, and 72 h, NOS inhibition significantly increased middle ear effusion in all the groups. However, cell infiltration into the middle ear cavity and hyperplasia of the middle ear mucosa were unaffected by L-NAA administration. This study suggests that NO may be involved in regulating vascular permeability, serum leakage into the middle ear mucosa, and/or extracellular fluid movement through the middle ear mucosal epithelium [44].

#### 3.4.4. MDA

To investigate the role of NO, free oxygen radicals, and antioxidants in the development of tympanosclerosis in chronic otitis media patients, a study was conducted with 65 patients who underwent tympanoplasty or tympanoplasty with mastoidectomy. The patients with tympanosclerotic plaques in the tympanic membrane, middle ear mucosa, ossicular chain, or mastoid bone were classified as group 1 (*n* = 34), and those without plaques as group 2 (*n* = 31). NO and MDA levels in samples from the middle ear mucosa (*p* = 0.001) and tympanic membrane (*p* = 0.01), along with plasma MDA activity levels, were higher in group 1 than in group 2. Moreover, erythrocyte catalase activity levels were significantly lower in group 2 compared to group 1 (*p* = 0.001). These results suggest that NO, free oxygen radicals, and catalase may play a role in the development of tympanosclerosis in chronic otitis media patients [31].

#### 3.4.5. LPS and L-NAME

To investigate the association with mucus production in chronic otitis media with effusion, Sprague–Dawley rats were used to create a model of otitis media with effusion. The role of NO in mucin secretion from the middle ear epithelium was examined using N-nitro-L-arginine methyl ester (L-NAME), a competitive inhibitor of NOS. After 7 days, the volume of effusion and the amount of collected mucus were significantly greater in the ears exposed to LPS than in the controls, showing mucous cell hyperplasia. Mucin production and mucous cell hyperplasia were inhibited in the ears treated with LPS and L-NAME. These results indicate that NO is associated with mucus secretion in chronic otitis media with effusion [32].

Another study explored the role of NO and peroxynitrite in mucociliary activity in OME experiments. Twenty guinea pigs were divided into one control group and three experimental groups: LPS group, N(G)-nitro-L-arginine methyl ester (L-NAME) group, and uric acid (UA) group. Dye transfer time was significantly delayed in the LPS group compared to the control group and significantly reduced in the L-NAME and UA treatment groups (*p* < 0.01). Histopathological examination showed reduced inflammation and mucosal thickening in the treatment groups compared to the LPS group, but these results were not statistically significant. Immunoreactivity for 3-NT was strong in the LPS group and decreased in the treatment groups (*p* < 0.05). Therefore, it was reported that LPS induced mucociliary dysfunction in the middle ear via NO and peroxynitrite-mediated pathways [33].

Studies claiming that NO is involved in the pathogenesis of otitis media have assessed the effects of NO donors and NOS inhibitors; their experimental results are summarized in Figure 3. An increase in NO expression through the administration of NO donors such as ISDN and SNAP was found to worsen the symptoms of otitis media. In contrast, a reduction in NO expression through the administration of NOS inhibitors such as ONO-17, L-NAME, and uric acid was found to improve the symptoms of otitis media.

#### 3.4.6. MPO, 4HNE, and L-NAME

In a study involving 61 COM patients and 30 controls, serum myeloperoxidase (MPO) activity, 4-hydroxynonenal (4-HNE), MDA, total antioxidant capacity (TAC), and NO were compared between the two groups. The COM patients were divided into two groups based on the presence of cholesteatoma (with cholesteatoma, *n* = 21; without cholesteatoma, *n* = 40). Serum MPO activity and levels of 4-HNE, MDA, and NO in the COM patients were significantly higher than those in the control group (all *p* < 0.001), while TAC levels were significantly lower (all *p* < 0.001). In the patients with cholesteatoma, serum MPO activity and levels of MDA, 4-HNE, and NO were significantly higher, and TAC levels were significantly lower than in the patients without cholesteatoma, though the differences between the groups were not statistically significant. These results suggest that increased oxidative stress in COM patients may be related to decreased antioxidant levels [34].

#### 3.4.7. NO, MPO, and CAT

To investigate the effects of oxidative stress and antioxidant status in children with chronic otitis media with effusion (COME) and acute otitis media (AOM), a study was conducted with a total of 107 children aged 2 to 13 years divided into 31 AOM patients, 39 COME patients, and 37 controls, from whom venous blood samples were collected. MPO, NO, and CAT levels were significantly higher in the AOM and COME groups compared to the control group (*p* = 0.040, *p* = 0.001, *p* = 0.044). This study observed the activity of antioxidants and oxidative stress in children with COME and AOM [35].

### 3.5. Clinical Implications of NO

Research into the clinical applications of NO-based therapy is ongoing. The efficacy of artificially supplied NO provided by NO donors has been found to depend on the concentration profile of NO in the disease area compared with the rest of the body. At low concentrations, NO interacts with transition metal-containing proteins, including those with heme groups and metal regulatory transcription factors, to regulate various biological processes and disease progression. In addition to having direct effects on biomolecules, NO can produce biologically active intermediates and reactive nitrogen species, such as nitrogen dioxide, at high concentrations [45]. Picomolar levels of NO contribute to angiogenesis and cell proliferation [46]. Because NO has an extremely short half-life, its delivery must be highly targeted and selective. Nano-delivery systems for NO are therefore being investigated [47].

Conversely, because NO inhibitors have potential side effects, NO inhibition is rarely used in clinical treatments. Inhibiting NO may be detrimental, particularly for patients with cardiovascular and renal diseases [48]. NO inhibition may lead to endothelial dysfunction and inflammatory syndrome, increasing cardiovascular morbidity and mortality [49]. Additionally, NO inhibition in patients with chronic kidney disease could worsen vasoconstriction, elevate blood pressure, and exacerbate atherosclerosis, thereby accelerating the progression of kidney disease [50,51]. Currently, the most common method of inhibiting NO is through the administration of synthetic NOS inhibitors. Despite their potential side effects, drugs such as N-nitro-L-arginine methyl ester (L-NAME) have been shown to have therapeutic benefits. For example, L-NAME can treat septic shock by maintaining blood pressure [52], and chronic L-NAME treatment has been found to suppress tumor growth by reducing angiogenesis, migration, and invasiveness [53]. Inhibition of nNOS has anticonvulsive properties [54], and inhibition of iNOS in mice can attenuate graft-versus-host disease by decreasing hematopoietic indices and concomitant susceptibility to bacterial infection [55].

### 3.6. Synthesis of NO in Otitis Media

**Figure 4:**
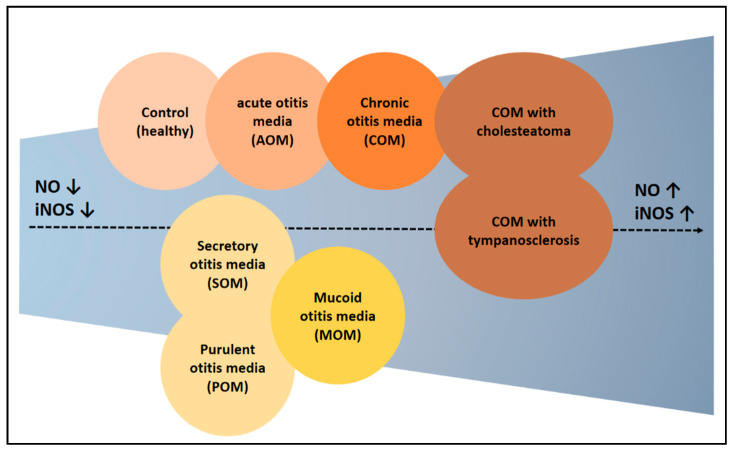
Experimental results claiming high expression of NO in otitis media. NO levels were higher in the otitis media patients compared to the control. Additionally, there were differences in NO expression depending on the type of otitis media. Generally, chronic otitis media showed higher NO levels. The groups on the right show a progressively higher NO expression. ↑ means increase, ↓ means decrease.

The review process has yielded several meaningful insights. First, the probability of elevated NO expression was higher in patients with chronic otitis media than in other types of otitis media (Figure 4). Notably, NO expression was higher in patients with cholesteatoma than in those without it, suggesting the need to determine whether the mechanism underlying the formation of cholesteatoma is related to NO. Second, NO is regarded as a substance with dual effects, providing therapeutic benefits through both donation and inhibition. Results showing that NO contributes to the pathogenesis of otitis media suggest that the latter approach may be more appropriate, particularly in chronic otitis media, in which NO expression is likely to be abnormally high. Because NO has been shown to inhibit infection through biofilm formation, NO donors may be beneficial in patients with acute otitis media, in which infection is a decisive factor. Taken together, these results suggest that acute otitis media caused by infection should be treated with NO donors, whereas chronic otitis media with an abnormally high NO expression should be treated with NO inhibitors.

Due to the dual effects of NO, research on therapeutic methods has yielded conflicting results. This suggests the need for in-depth exploration of confounding factors (such as the type of otitis media, sample type, and targeted substances) that hinder consistent research outcomes. If the role of NO in otitis media is dependent on the duration of disease, it would be important to examine the effects of disease duration on changes in the role of NO. Additionally, methods are needed to precisely control the location and concentration of applied NO, making clinical application of NO possible in the treatment of otitis media.

## 4. Conclusions

Research on animals and humans has demonstrated that NO, nNOS, eNOS, and iNOS play significant roles in the development of otitis media. Although all the studies reviewed analyzed the impact of NO on otitis media, their experimental results and conclusions varied. Factors that may have contributed to these discrepancies include the type of otitis media, the duration of the condition, the types of samples collected, and the types of targeted NOS. Factors contributing to differences in research results included the type of otitis media, the use of normal controls, research subjects, experimental methods, selection of control groups, types of samples collected, types of NOS, NOS stimulants, and NOS inhibitors. Most of the studies found that NO levels were higher in the patients with acute otitis media (AOM), otitis media with effusion (OME), chronic otitis media (COM), COM with cholesteatoma, and COM with tympanosclerosis than in the controls. Increases in NO, NO stimulants, NO metabolites, and NO biomarkers were associated with increases in the incidence of otitis media, as well as with worsened symptoms and pathophysiology of the disease. Conversely, administering NO inhibitors improved the symptoms and pathophysiology of otitis media.

Other studies, however, contradict this conclusion. For example, low levels of NO may be beneficial in treating patients with otitis media caused by *S. pneumoniae* infection. Low concentrations of NO can inhibit the formation of bacterial biofilms, thereby reducing the likelihood of infection and enhancing bacterial eradication and antibiotic efficacy. Although NO may have a dual role in the treatment of otitis media, a synthesis of the current literature suggests that NO is more likely to be associated with the occurrence and pathophysiology of otitis media. NO may therefore play contrasting roles in acute otitis media caused by infections, as well as in chronic otitis media with an abnormal NO overexpression.

## Figures and Tables

**Table 1:** Three NOS and their characteristics and biological functions.

NOS	NOS Isoform	Alternative Description	Human Chromosome	Molecular Mass (kDa)	Distinctive Properties	Subcellular Localization	Tissue Expression	Features	Primary Site	Main Function
NOS-I (nNOS)	Neuronal	Type I nNOS ncNOS bNOS	12	160	Ca^++^-dependent, constitutively expressed	Binds to specific proteins via an N-terminal PDZ domain	Neuronal cells Skeletal muscle	Constitutive, Ca^++^/CaM-dependent	1. Neuronal tissues 2. Skeletal muscle 3. Lung epithelium	1. Neurotransmission 2. Relaxation of vascular and non-vascular smooth muscle
NOS-II (iNOS)	Inducible	Type II iNOS macNOS	17	130	Ca^++^-independent, induced by inflammatory stimuli (cytokines, LPS)	Soluble?	Macrophages Hepatocytes Astrocytes Smooth muscle cells (and many more)	Inducible, Ca^++^-independent	1. Astrocytes 2. Chondrocytes 3. Dendritic cells 4. Epithelial cells 5. Fibroblasts 6. Macrophages 7. Osteoclasts 8. Various cancer cells	1. Cytotoxicity 2. Host defense
NOS-III (eNOS)	Endothelial	Type III eNOS ecNOS	7	134	Ca^++^-dependent, constitutively expressed	Targets to the Golgi and the caveolae via N-terminal myristoylation and palmitoylation	Endothelial cells Epithelial cells Cardiomyocytes	Constitutive, Ca^++^/CaM-dependent, membrane-associated	1. Endothelial cells 2. Cardiac myocytes 3. Hippocampal pyramidal cells	1. Platelet aggregation 2. Vascular tone maintenance 3. Angiogenesis 4. Corpora cavernosa relaxation 5. Smooth muscle proliferation control

**Table 2:** Studies claiming that NO worsens otitis media.

Author [Reference]	Study Design	Species and/or Sample	Types of Otitis Media	Detection Method	Target gene(s) or Pathway(s) Associated with NOS	Results/Conclusions
Fan et al. (2019) [19]	Human study	50 children with OME and 50 controls	Otitis media with effusion	NO levels were determined using ELISA (enzyme-linked immunosorbent assay)	NO	In the observation group, the percentage of NO levels in peripheral blood was significantly greater than that in the control group (*p* < 0.01). Furthermore, within the observation group, NO levels in the MEE were markedly higher than those found in peripheral blood (*p* < 0.01). In children with OME, there is an elevation in peripheral blood NO levels. These measurements offer valuable insights for diagnosing OME in pediatric patients.
John et al. (2001) [20]	Human study	55 patients	Otitis media with effusion	Colorimetric assay (Griess method)	NO	In human MEE, the concentrations of NO₂⁻/ NO₃⁻ were found to be highest in cases of mucoid otitis media, followed by serous and then purulent otitis media. Many children who continue to have effusion after acute OM treatment progress to MOM, which is considered the most advanced stage within the OME spectrum. The elevated NO levels observed in MOM further imply that NO might play a role in mediating mucin secretion during the development of MOM.
Capper et al. (2003) [21]	Human cell line study	HT-29MTX goblet cells	Otitis media with effusion	Mucin analysis (ELISA assay)	NO	When ISDN, an NO donor, is added to the cell culture medium containing the goblet cell line HT29-MTX, there is an observed increase in the production of human MUC5AC mucin. The peak stimulation is achieved after 1 h of NO donor exposure, resulting in a 30% increase in mucus production compared to baseline levels. Additionally, the increase in mucus production is dose-dependent on the concentration of ISDN. The administration of NO via ISDN leads to an increase in mucus production that is related to both dose and time. This finding further supports an inflammatory model for mucus secretion in OME.
Martin et al. (2004) [22]	Animal study	22 chinchillas	Mucoid otitis media (MOM)	Auditory brainstem response, histopathologic study, mucin analysis (periodic acid–Schiff method)	NO	Mucin levels were significantly elevated in the LPS + SNAP (NO donor) group compared to the group treated with LPS alone. After 96 h, both the LPS and LPS + SNAP groups showed thickened mucosa accompanied by subepithelial edema, focal hemorrhage, and hyperemia. These findings bolster the hypothesis that exogenous NO can enhance mucin production in LPS-induced OM and exacerbate the severity of inflammation in the middle ear mucosa. The study indicates that NO may play a role in the progression of MOM from SOM in the pathogenesis of OM.
Lee et al. (2013) [23]	Human study	96 children with OME	Otitis media with effusion	The level of iNOS mRNA in middle ear effusion was assessed using real-time polymerase chain reaction	iNOS	Overall, the patients who were culture-positive exhibited higher levels of NOS mRNAs compared to those who were culture-negative. However, this difference did not reach statistical significance. NOS plays a cooperative role in the innate immune response and is closely linked to OME.
Başoğlu et al. (2011) [24]	Animal study	16 rabbits	Gastric content-induced middle ear inflammation	Histochemical staining, immunohistochemical staining, H&E staining, light microscopy	iNOS, eNOS	The expression level of iNOS showed a significant difference between the experimental and control groups, whereas the expression level of eNOS did not differ significantly between these groups. This study illustrates that middle ear inflammation induced by gastroesophageal reflux is linked to an increased expression of iNOS.
Long et al. (2003) [25]	Animal study	55 Sprague–Dawley rats	Pneumococcal otitis media	Real-time PCR, ELISA	iNOS	The transparent variant of *S. pneumoniae* is a stronger inducer of inflammation, leading to the accumulation of more inflammatory cells and significantly higher increases in the expression and production of inflammatory mediators. The opacity variants of *S. pneumoniae* affect the timing of mRNA expression for inflammatory mediators in the middle ear.
Forséni et al. (2001) [26]	Human study	9 biopsy specimens from children with secretory otitis media, 11 biopsy specimens from patients with chronic otitis media and tympanosclerosis	Secretory otitis media, chronic otitis media	Immunohistochemical technique	iNOS	Biopsy specimens from the children with secretory otitis media exhibited more positive staining for macrophages, B cells, and IL-6 compared to those from patients with chronic otitis media and tympanosclerosis. Conversely, biopsy specimens from the patients with chronic otitis media and tympanosclerosis more frequently showed positive staining for iNOS than those from secretory otitis media. In patients with secretory otitis media, an early phase of the disease, macrophages, B cells, and IL-6 were labeled more frequently. In contrast, iNOS was more frequently observed in patients with tympanosclerosis, indicating a later phase of the disease.
Yilmaz et al. (2006) [27]	Animal study	12 guinea pigs (6 with experimental OME and 6 controls)	Otitis media with effusion	Erythrocyte sediments, NOS activity assay, erythrocyte NO levels measured using the Griess reagent	NOS, NO	In experimental otitis media with effusion, the NOS activity and NO levels were significantly higher compared to the control group. In guinea pigs with experimental OME, erythrocytes exhibit an increased NOS activity and elevated NO levels. These heightened NO levels may significantly contribute to cell and tissue damage associated with experimental otitis media with effusion.
Sone et al. (2003) [28]	Animal study	16 Sprague–Dawley rats	Damage to the cochlear lateral wall induced by endotoxin (lipopolysaccharide)-induced otitis media	Measurement of the cochlear blood flow using a laser Doppler flowmeter, electron microscope examination	NO	ONO-1714, a NOS inhibitor, is significantly more effective than the steroid dexamethasone in combating LPS-induced disorders of the cochlear wall. In rats treated with ONO-1714, cochlear blood flow normalized and increased more following prostaglandin E1 application compared to those treated with PBS. These drugs can help protect the function of the spiral ligament during acute otitis media. The intratympanic administration of a NOS inhibitor proved effective in treating disturbances in the cochlear lateral wall caused by otitis media.
Pudrith et al. (2010) [29]	Animal study	53 chinchillas	Otitis media with effusion	Griess reagent	NO	In an LPS-induced experimental OME model in chinchillas, glucocorticoids reduced NO levels in middle ear effusion. While treatment with glucocorticoids at 0.1% concentrations did not consistently lead to significant reductions in NO, a 1% concentration of glucocorticoids significantly reduced NO concentration by 55.3%. Glucocorticoids have the potential to lower the levels of NO, a potentially ototoxic substance, in middle ear effusion. Nonetheless, further research, including clinical trials, is necessary to ascertain if this effect is applicable in clinical practice.
Hebda et al. (2002) [30]	Animal study	72 Sprague–Dawley rats	Acute otitis media	RT-PCR	iNOS	During AOM, the expression of messages for cytokines and iNOS is downregulated by corticosteroid treatment. In rats with AOM, tacrolimus treatment led to decreased messenger levels for iNOS and the cytokines tested, with effects similar in magnitude to those seen with dexamethasone treatment. The extensive impact of dexamethasone and tacrolimus on cytokine and iNOS messenger expression indicates that these treatments may exert strong anti-inflammatory effects in AOM.
Karlıdağ et al. (2004) [31]	Human study	65 patients	Chronic otitis media with and without tympanosclerosis	Griess reaction, modified cadmium reduction method, spectrophotometric method	NO, MDA	In specimens taken from the middle ear mucosa, tympanic membrane, and correspondingly from plasma, the NO and MDA levels were higher in group 1 (patients with tympanosclerotic plaques) compared to group 2 (patients without tympanosclerosis). Additionally, group 1 exhibited lower antioxidant activity levels (superoxide dismutase and catalase) in their erythrocytes than group 2. NO, free oxygen radicals, and catalase might play a role in the development of tympanosclerosis in patients with chronic otitis media.
Rose et al. (1996) [32]	Animal study	20 Sprague–Dawley rats	Otitis media with effusion	ELISA, immunohistochemistry	NOS	After 7 days, the LPS-exposed ears had a significantly greater volume of effusion and amount of collected mucin compared to the controls. Additionally, antimucin immunostaining revealed mucous cell hyperplasia as a response to LPS exposure. Treatment with L-NAME inhibited the LPS-induced production of mucin and mucous cell hyperplasia in the ears. LPS triggers the hypersecretion of mucin in OME, and inhibiting NO synthesis prevents the LPS-induced production of mucin in middle ear effusion.
Jeon et al. (2006) [33]	Animal study	20 guinea pigs	Otitis media with effusion	5% Coomassie Brilliant Blue dye transfer time, light microscopy, immunohistochemistry	NO	In the LPS group, dye transfer time was significantly delayed compared to the control group; conversely, it was notably shortened in the groups treated with L-NAME (NOS inhibitor) or uric acid (peroxynitrite scavenger). Histopathological examination revealed reduced inflammation and mucosal thickening in these treated groups compared to the LPS group. NO plays a role in every stage of OM pathogenesis, and its harmful effects may be mediated through the formation of RNS. New therapeutic strategies could be developed to mitigate the potential role of NO and peroxynitrite. However, given their defensive role against invading organisms, the timing of administration must be carefully considered.
Garça et al. (2013) [34]	Human study	61 patients with COM and 30 controls	Chronic otitis media, COM with cholesteatoma (CCOM)	Serum TAC levels were measured spectrophotometrically; serum NO levels were measured using the Griess reagent	Lipid peroxidation, MAD, MPO, NO, TAC	Our study revealed that the patients with COM had significantly elevated serum NO levels and reduced TAC levels compared to the healthy controls. Furthermore, serum NO levels were markedly higher in the patients with cholesteatoma than in those without, whereas TAC levels were significantly lower. Enhanced oxidative stress appears to be linked with reduced antioxidant levels in patients with COM, suggesting that increased oxidative stress may contribute to the pathogenesis of COM.
Sagiroglu et al. (2018) [35]	Human study	107 children	Chronic otitis media with effusion (COME), acute otitis media (AOM)	MPO activity was determined using the O-dianisidine method; CAT activity was assayed using Beutler’s method; NO levels were measured using the Griess reagent	MPO, CAT, NO	The levels of MPO, CAT, and NO were significantly elevated in the COME and AOM groups compared to the controls. CAT levels were slightly higher in the COME patients than in those with AOM. Serum levels of oxidative stress markers and antioxidant products actively contribute to the pathogenesis of COME and AOM. We believe these findings are crucial for the diagnosis and treatment of patients.
Tong et al. (2008) [36]	Animal study	80 Sprague–Dawley rats	Acute otitis media	Real-time PCR, ELISA, H&E staining	NO	Nonviable NTHi (nontypeable *Haemophilus influenza*) 2019 parent strain induced a significant upregulation of iNOS gene expression compared to the B29 (NTHi LOS htrB gene mutant group) cohort. This study indicates that the disruption of the NTHi LOS htrB gene may impact the temporal mRNA expression of inflammatory mediators within the middle ear.

Abbreviations: OME, otitis media with effusion; iNOS, inducible nitric oxide synthase, COM, chronic otitis media; CCOM, COM with cholesteatoma; NO, nitric oxide; TAC, total antioxidant capacity; MDA, malondialdehyde; MPO, myeloperoxidase; PCR, polymerase chain reaction; eNOS, endothelial nitric oxide synthase; PBS, phosphate-buffered saline; ELISA, enzyme-linked immunosorbent assay; L-NAME, N-nitro-L-arginine methyl ester; MEE, middle ear effusion; MOM, mucoid otitis media; SOM, serous otitis media; NTHi, nontypeable *Haemophilus influenzae*; LOS, lipooligosaccharide; mRNA, messenger ribonucleic acid; H&E, hematoxylin and eosin; LPS, lipopolysaccharide; COME, chronic otitis media with effusion; AOM, acute otitis media; CAT, catalase; SNAP, S-nitroso-N-acetylpenicillamine; MOM, mucoid otitis media; SOM, serous otitis media; ISDN, isosorbide dinitrate; RNS, reactive nitrogen species; LOS, lipooligosaccharide.

**Table 4:** The study did not clearly state whether NO worsens or helps treat otitis media.

Author [Reference]	Study Design	Species and/or Sample	Types of Otitis Media	Detection Method	Target gene(s) or Pathway(s) Associated with NOS	Results/Conclusions
Ates et al. (2017) [43]	Animal study	89 patients	Otitis media with effusion	Detection of the Clu298Asp polymorphism in eNOS was achieved by PCR-RFLP	eNOS	There was no statistically significant difference in genotypic distributions (Glu/Glu (G/G), Glu/Asp (G/T), Asp/Asp (T/T)). However, when comparing allele distributions, a significant relationship was found, with the G allele identified as a predisposing factor for genetic susceptibility to the development of OME. The G allele was identified as a predisposing factor for genetic susceptibility to the development of OME. Further comprehensive research is needed to explore new diagnostic and treatment modalities for OME, taking into account the eNOS polymorphism in pediatric patients.
Ryan et al. (2001) [44]	Animal study	32 Hartley guinea pigs	Immune-mediated otitis media	Evaluation of effusion, H&E staining, light microscopy	NOS	Inhibition of NOS with NG-amino-L-arginine (L-NAA) led to a significant increase in middle ear effusion across all three time periods. This increase was prevented by adding excess L-arginine, which counteracts the inhibitory effects of L-NAA. The results indicate that NO plays a role in regulating the permeability of middle ear vasculature, the transudation of serum into the middle ear mucosa, and/or the movement of extracellular fluid across the middle ear mucosal epithelium.

Abbreviation: OME, otitis media with effusion; eNOS, endothelial nitric oxide synthase; NO, nitric oxide; H&E, hematoxylin and eosin; L-NAA, NG-amino-L-arginine; PCR, polymerase chain reaction; RFLP, restriction fragment length polymorphism.
